# Cgaln: fast and space-efficient whole-genome alignment

**DOI:** 10.1186/1471-2105-11-224

**Published:** 2010-04-30

**Authors:** Ryuichiro Nakato, Osamu Gotoh

**Affiliations:** 1 Department of Intelligence Science and Technology, Graduate School of Informatics, Kyoto University, Yoshida-Honmachi, Sakyo-ku, Kyoto-shi, Kyoto 606-8501, Japan; 2 Institute of Molecular and Cellular Biosciences, The University of Tokyo, 1-1-1, Yayoi, Bunkyo-ku, Tokyo, 113-0032, Japan; 3 National Institute of Advanced Industrial Science and Technology, Computational Biology Research Center, 2-42 Aomi, Koto-ku, Tokyo 135-0064, Japan

## Abstract

**Background:**

Whole-genome sequence alignment is an essential process for extracting valuable information about the functions, evolution, and peculiarities of genomes under investigation. As available genomic sequence data accumulate rapidly, there is great demand for tools that can compare whole-genome sequences within practical amounts of time and space. However, most existing genomic alignment tools can treat sequences that are only a few Mb long at once, and no state-of-the-art alignment program can align large sequences such as mammalian genomes directly on a conventional standalone computer.

**Results:**

We previously proposed the CGAT (Coarse-Grained AlignmenT) algorithm, which performs an alignment job in two steps: first at the block level and then at the nucleotide level. The former is "coarse-grained" alignment that can explore genomic rearrangements and reduce the sizes of the regions to be analyzed in the next step. The latter is detailed alignment within limited regions. In this paper, we present an update of the algorithm and the open-source program, Cgaln, that implements the algorithm. We compared the performance of Cgaln with those of other programs on whole genomic sequences of several bacteria and of some mammalian chromosome pairs. The results showed that Cgaln is several times faster and more memory-efficient than the best existing programs, while its sensitivity and accuracy are comparable to those of the best programs. Cgaln takes less than 13 hours to finish an alignment between the whole genomes of human and mouse in a single run on a conventional desktop computer with a single CPU and 2 GB memory.

**Conclusions:**

Cgaln is not only fast and memory efficient but also effective in coping with genomic rearrangements. Our results show that Cgaln is very effective for comparison of large genomes, especially of intact chromosomal sequences. We believe that Cgaln provides novel viewpoint for reducing computational complexity and will contribute to various fields of genome science.

## Background

Sequence alignment is one of the most fundamental approaches in bioinformatics. It finds common subsequence patterns shared by the input sequences, and this information supports the identification of evolutionarily conserved genes or other functional regions, the prediction of 1-3D structures of proteins and RNAs, and the analysis of evolutionary relationships between the species [1]. With the rapid increase in genomic sequence data in recent years, there is great demand for alignment programs that can allow direct comparisons of whole genomic sequences. Cross-species genomic sequence comparison reveals homologous DNA segments and ancestral rearrangements [2,3], while intra-species genomic comparisons (e.g., human-human) can identify individual differences, such as SNPs, indels, copy number variations, and other types of peculiarities [4,5].

Optimal pairwise alignment can be obtained by a dynamic programming (DP) algorithm with *O*(*L*^2^) time and *O*(*L*) space, where *L *is the length of an input sequence [6]. However, even bacterial genomic sequences often exceed 1 Mb in length, thus prohibiting the application of full-blown DP. Most existing alignment algorithms apply fast seed-search algorithms, such as suffix trees, suffix arrays, and hash tables, to extract high-scoring pairs (HSPs) of subsequences from the input sequences [7]. Recent efforts have developed some alignment tools for genomic sequences, which include global alignment programs such as GLASS [8], AVID [9], and LAGAN [10], as well as local alignment programs such as Blastz [11], CHAOS [12], MUMmer [13], and WABA [14]. Though these programs can align small genomic sequences such as those of bacteria, the comparison of large genomic sequences such as those of mammals still requires large amounts of time and memory. For example, Blastz succeeded in the whole-genome alignment of human and mouse by 481 days of CPU time and a half day of wall clock time on a cluster of 1024 833 MHz CPUs [11]. The Berkeley Genome Pipeline [15] also reported a whole-genome human-mouse comparison by using AVID based on the outputs of BLAT [16] in 17 hours on a cluster of 16 2.2 GHz CPUs (20 CPU d).

This procedure was later expanded to human-mouse-rat alignment with LAGAN [17]. However, these kinds of genome comparisons on a standalone computer remain difficult because of the long computational time and the large amount of memory necessary for computation, even on the much-improved hardware systems presently available.

Although global alignment typically has higher sensitivity than local alignment for less-similar sequences such as noncoding regions [18], it should be applied to consistently homologous regions, and hence it cannot treat rearrangements of genomes. As a genomic sequence pair generally has rearrangements or unrelated regions, global alignment is not appropriate for whole-genome comparison in most cases. Local alignment can treat rearrangement but is likely to give "noisy" outputs; if there are unmasked repetitive regions in the input sequences, a lot of alignments can be generated, most of which may not be interesting for general users. Shuffle-LAGAN (SLAGAN), a "glocal" alignment method, was developed to overcome the shortcomings of local and global alignment by identifying the breakpoints of rearrangements [19]. However, the problem of computational complexity remains poorly resolved.

Moreover, these existing tools require the splitting up of input genomic sequences into short chunks because of the limitation in available computer memory. For human-mouse comparison, Blastz divided the human genome into 3000 segments of about 1 Mb in length with a 10 Kb overlap, while AVID and SLAGAN split the mouse genome into chunks of 250 Kb in length. Such splitting strategies have several disadvantages [20]. Namely, the manual splitting and uniting processes of the sequences and alignment are tedious, potentially invoking several types of errors. In particular, a splitting strategy may divide a homologous region into chunks and align them separately, such that a contiguous orthologous segment might be recognized as separate entities. Consequently, there remains no handy, practical method of aligning vertebrate-sized genomes for most researchers.

To overcome the computational difficulty, we previously proposed the basic idea of the CGAT (Coarse-Grained AlignmenT) algorithm [21]. This algorithm involves two levels of computation: block-level and nucleotide-level alignments. The former, "coarse-grained" local alignment step explores the genomic rearrangements and reduces the sizes of the regions to be aligned in the next step. The latter step is devoted to detailed global alignment within limited regions. By applying this algorithm to several bacterial genomes, we have shown that this two-step procedure can not only speed up computation but also facilitate noise reduction with consideration of genomic rearrangements. This procedure is a new strategy to overcome the disadvantages of global and local alignments, and thus differs from glocal alignment of SLAGAN.

In this paper, we report on an update of the CGAT algorithm and its associated program Cgaln, which both improves accuracy and lowers computational costs, and now allows the alignment of not only bacterial genomes but also whole mammalian genomes in a single run. (For simplicity, we hereafter use "Cgaln" to refer to both the updated algorithm and the program.) We quantitatively evaluated the performance of Cgaln in comparison with those of several other genomic alignment programs. The results show that Cgaln is as sensitive and specific as Blastz, which is shown to perform the best among the existing programs. Cgaln runs several times faster and is considerably more memory-efficient than Blastz with a tuned set of parameters. Cgaln can now regulate the outputs of repetitive alignments; under special conditions, all repetitious outputs other than the best-scored ones are suppressed. This option is useful for detecting SNPs and small indels along an orthologous genomic sequence pair.

The Cgaln source code is freely available at http://www.genome.ist.i.kyoto-u.ac.jp/~aln_user/cgaln/.

## Methods

### Overview

**Figure 1:**
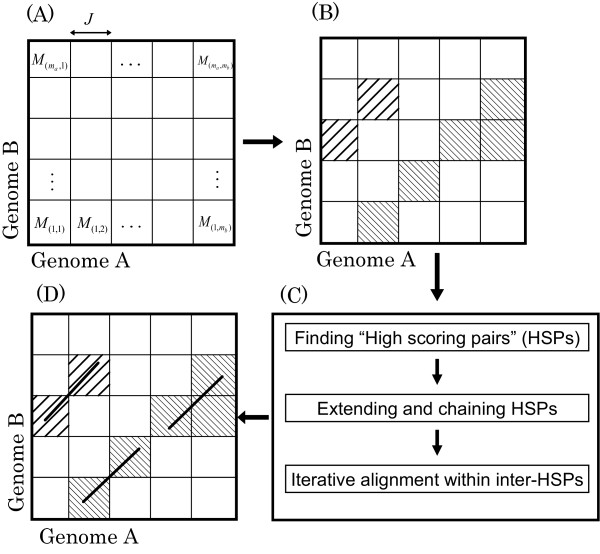
**The flow of the Cgaln algorithm**. *J *indicates the length of a block. *M*_*x,y *_indicates the similarity score between blocks. (A) and (B) show the block-level alignment, (C) shows the flow of nucleotide-level alignment, and (D) shows the alignment result. The cells with oblique lines indicate aligned block cells, and the diagonal lines in (D) indicate the aligned subsequences at the nucleotide level.

Figure 1 shows the flow of Cgaln. First, Cgaln divides the input sequences into "blocks" with a fixed length (Figure 1A). These blocks are taken as "elements" to be aligned at the block level. Each cell of the meshlike structure is associated with a block-to-block similarity score. The similarity between two blocks, each from the two input sequences, is evaluated by the frequency of seeds (*k*-mers) commonly found in the blocks. Similar methods based on seed counts have been used to quickly estimate the degree of similarity between two protein sequences [22,23]. Based on these similarity scores, the block-level alignments are obtained by a local DP algorithm (Figure 1B). For the local DP, we apply the Smith-Waterman algorithm [24] modified so that sub-optimal similarities are also reported [25]. The size of a block is arbitrary within the limits that both the number of blocks and the size of each block should be < 2^16 ^in order to be represented by unsigned short integers.

The nucleotide-level alignment is conducted on the restricted regions included in the block-level alignment found in the first stage (Figure 1C). We adopt a seed-extension strategy widely used in homology search programs such as Blast [26]. The obtained seed matches are integrated into gapless HSPs. The HSPs are then filtered and chained to conform to coherent alignment. If necessary, the chained HSPs may serve as anchor points, the subsequences between which are aligned by a standard DP algorithm or recursive search for shorter seeds.

The above-mentioned algorithm implicitly assumes that genomic rearrangements smaller than the block size of Cgaln (about 10 Kb) are rare, and they are not searched for with the default settings. To discover rearrangements smaller than the block size, we prepared the "-sr" option that considers inversions as small as a few hundred bases at the recursive phase applied to inter-HSP regions (see below).

Cgaln accepts two single- or multi-fasta files. When either or both files are in multi-fasta form, Cgaln aligns every pair of single-fasta entry sequences and outputs the united results. In this section, we assume for simplicity that both input sequences are single-fasta files. By setting the "-r" option, Cgaln examines both orientations of a sequence in a single run.

### Seed designing

Cgaln uses the "spaced seed" proposed in PatternHunter [27] for fast and sensitive seed matching. A *k*/*w *spaced seed is a discrete series of nucleotides of length *w *in which *k *<*w *positions are examined for nucleotide matching. Cgaln uses an 11/18 spaced seed by default, with the same pattern as that used in PatternHunter (expressed as 111*1**1*1**11*111, where "1" and "*" respectively indicate the positions to be examined or ignored). This pattern was designed to be most sensitive for a pair of randomly generated sequences with 70% nucleotide identities. Although some other seed patterns are more sensitive than this pattern under some conditions (e.g., for coding regions), we chose this seed design so that Cgaln could be generally applicable to whole genome sequences of various species. Optionally, a 12/19 or 13/20 spaced seed can be used, as suggested by Mak and Benson [28] (expressed as 1111*1*1**11**1*111 and 1111*1**11**11*1*111, respectively). We use the term *k*-mer hereafter to refer to these *k*/*w *spaced seeds, as the value for *k *is most important. A larger *k *is appropriate for longer sequences but requires more memory, and hence there is a compromise in the choice of the best value for *k*. The "code" of each seed is its quaternary expression calculated from the *k *examined positions by converting nucleotides A, C, G, and T into numerals 0, 1, 2, and 3, respectively. Thus, the code of a *k*/*w *seed is between 0 and 4^*k *^- 1.

### Block-level alignment (BA)

In this subsection, we describe block-level alignment (abbreviated as BA hereafter).

#### Measuring the score between two blocks

Let us denote the given input genomic or chromosomal (single-fasta) sequences *G*_*a *_and *G*_*b*_. Let *L*_*a *_and *L*_*b *_be the lengths of *G*_*a *_and *G*_*b*_, respectively. First, Cgaln divides *G*_*a *_and *G*_*b *_into blocks with the fixed length of *J*, except that the last block may be shorter than *J*. The numbers of blocks in *G*_*a *_and *G*_*b *_are denoted as *m*_*a *_and *m*_*b*_, and thus *m*_*a *_= ⌈*L*_*a*_/*J*⌉ and *m*_*b *_= ⌈*L*_*a*_/*J*⌉. Let  be the *x*-th block of *G*_*a *_and  be the *y*-th block of *G*_*b *_(1 ≤ *x *≤ *m*_*a*_, 1 ≤ *y *≤ *m*_*b*_).

The score *M*_*x,y*_, the measure of similarity between  and , is defined as the summation of the scores of seeds commonly found in both  and . The score of seed *k*_*i *_is evaluated by the probability of the chance of finding one or more matches of *k*_*i *_in a block pair. Let  be the total number of *k*_*i *_in *G*_*a*_, then the expected mean number of occurrences of *k*_*i *_in a block  is(1)

We assume the Poisson distribution for the probability of *k*_*i *_occurring *f*_*i *_times in a block , i.e.,(2)

*p*_*b*_(*f*_*i*_) is obtained analogously. When *k*_*i *_occurs *f*_*i *_times in  and *h*_*i *_times in , *k*_*i *_matches *f*_*i*_*h*_*i *_times between  and . It is clearly an overestimation to use this number as the measure of similarity between  and , because true homologous matches should line up around a single diagonal. In such a case, we regard min(*f*_*i*_, *h*_*i*_) as the number of matches as suggested by [23]. Thus, the score of seed *k*_*i *_is(3)

where the probability of seed *k*_*i *_occurring more than or equal to *x *times in a block is(4)

Summing up *s*(*k*_*i*_) for all *k*-mers, we obtain the similarity score as:(5)

#### Local DP alignment at block level

After obtaining *M*_*x,y *_for (1 ≤ *x *≤ *m*_*a *_and 1 ≤ *y *≤ *m*_*b*_), the local alignment of BA is conducted by using local DP as follows:(6)

where *d *is the gap penalty. In practice, *M*_*x*, *y *_is calculated in the process of this DP procedure to save the required memory from *O*(*m*_*a*_*m*_*b*_) to *O*(min(*m*_*a*_, *m*_*b*_)). To obtain the optimal and suboptimal locally best-matched alignments, we use the algorithm proposed by Gotoh [25]. This algorithm assigns each block cell to a "colony", which is a candidate for local alignment. To define the colony borders, we apply the X-drop-off approach [29]. At a certain block cell, if the score *F*_*x*, *y *_increases from 0 to a positive value, a new colony starts. A colony is "significant" if *F*_*x*, *y *_> *T*_*col *_somewhere in the colony, where *T*_*col *_denotes a predefined threshold. A colony ends when *F*_*x*, *y *_becomes ≤ 0 or falls by more than *T*_*col *_from the maximum score of the colony, then *F*_*x*, *y *_is reset to 0. Only significant colonies are retained for further analysis. This method can greatly reduce the storage requirement, while the computational time remains *O*(*m*_*a*_*m*_*b*_).

It should be noted that *M*_*x*, *y *_is always ≥ 0 even between nonhomologous blocks, while it is a prerequisite that, on average, the similarity score must be less than zero for the local DP algorithm to work properly [30]. Consequently, we subtract a constant bias *B *from each *M*_*x*, *y*_, so that , i.e., the X-drop-off should finish after passing through, on average, *a *unrelated cells (*a *= 5 by default). If there are repetitive sequences that escaped masking, BA may output them many times. Cgaln has an option for suppressing such repetitious outputs. If there are inconsistent colonies that overlap each other, the "-fc" option filters all of them out except for the one with the highest scored.

#### Tables of input sequences

BA uses four kinds of tables: a "seed table", an "index table", and two "Poisson tables". The seed table stores the number of occurrences of each seed *k*_*i *_in a genomic sequence, whereas the index table stores a list of blocks where each *k*_*i *_resides. The seeds occurring more than a certain number of times (1024 by default) are omitted in subsequent analyses as highly repetitive sequences. The two Poisson tables respectively store the probabilities that a fixed number of *k*_*i*_s (*p*(*x*_*i*_)) and more than a fixed number of *k*_*i*_s (*p*(≥ *x*_*i*_)) occur in a block, where the probabilities are calculated based on the Poisson distribution. The probabilities of occurrences exceeding an upper limit (3 by default) are ignored as repeats. It is sufficient to construct these tables only once for each genomic sequence and for each orientation. They are stored in binary files and read into memory at run time. By using these tables, seed matching on *G*_*a *_can finish in *O*(*L*_*a*_/4^*k*^) per k-mer in *G*_*b *_[31]. Thus, the similarity measure matrix, *M*_*x*, *y *_(*x *= 1.. *m*_*a*_, *y *= 1.. *m*_*b*_), is computed in *O*(*L*_*a*_*L*_*b*_/4^*k*^).

### Nucleotide-level alignment (NA)

**Figure 2:**
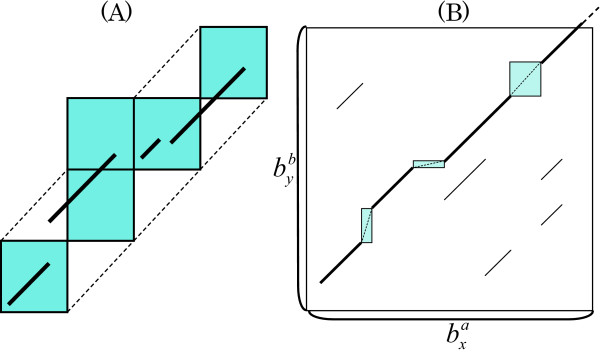
**The nucleotide-level alignment (NA)**. (A) Extended region for NA. The squares indicate aligned block cells, and the extended area is enclosed by dotted lines. (B) Chaining and integration of HSPs within an extended area (bold lines). Inter-HSP regions (rectangles) may be aligned with shorter seeds or global DP, while spurious HSPs (thin lines) are filtered out.

Cgaln applies the nucleotide-level alignment (abbreviated as NA hereafter) within the restricted areas that are composed of block cells included in the local alignments of BA. However, the area covered by the set of block cells thus obtained is insufficient for NA, because the expected results of NA may shift slightly away from the aligned cells. Hence, we extend the area to be searched by NA to the "envelope" of the block cells found in the first stage (see Figure 2A).

#### Generating and chaining HSPs

At NA, Cgaln adopts a seed-finding approach with the 11-mer spaced seed. Figure 2B shows NA within a cell. First, the seed matches are searched for by using an index table again. This index table stores the list of positions at the nucleotide level within the restricted region, while the index table for BA stores genome-wide positions at the block level. A group of matches are integrated into one larger matching segment if the matches are closer to each other than a given threshold (20 by default) with no gap (i.e., if they lie on the same diagonal in the dot matrix). We define such a gapless matching segment as an HSP. Lonesome matches, individual matches that are not integrated with any other matches, are filtered out. The score of each HSP is defined as usual as the sum of scores assigned to individual matches and mismatches included in it. The masked bases are omitted. Third, the HSPs are extended to both sides with no gap until the HSP score drops below a threshold. Finally, the extended HSPs are chained by computing a maximal-scoring collinear subset of them by a sparse-DP algorithm [32]. This step also helps to eliminate noisy HSPs. Cgaln penalizes a predefined value, *pen*_*overlap *_= max(overlap length - 10, 0) by default, for partially inconsistent (overlapping in either projection) HSP pairs. Cgaln may output some inconsistent but high-scoring HSPs. If one wants "unique" outputs, *e.g*. to detect SNPs, the "-cs" option of Cgaln increases this penalty to infinity and suppresses any outputs that are inconsistent with the highest-scoring alignment.

#### Iterative alignment within inter-HSP regions

Additionally, Cgaln aligns iteratively the regions between neighboring HSPs to improve sensitivity. First, the HSPs are extended with gaps toward both sides. We adopt DP with X-drop-off for this gapped extension. The second step varies with the length of the inter-HSP region. (1) If an inter-HSP region is shorter than the lower-threshold *T*_*low *_(50 bp by default), Cgaln uses standard global DP. (2) Or else, if it is below a higher-threshold *T*_*high *_(twice the block size by default), Cgaln applies the seed finding and chaining approaches again with shorter-spaced seeds (7-mer by default), and the interval regions are aligned by global DP if the longer interval is less than the given threshold *T*_*dp *_(200 bp by default). (3) If the inter-HSP region is longer than *T*_*high*_, that region is left unaligned.

At the iterative alignment step, Cgaln unmasks the repetitive sequences because there might be homologous regions in repetitive (masked) regions. By default, the DP match/mismatch scores of Cgaln are set to be identical to those of Blastz, derived by Chiaromonte *et al*. [33] with the gap open and extension penalties of 400 and 30, respectively.

### Evaluation method

It is difficult to evaluate the accuracy of genomic alignment because of the lack of "true" alignment data [34]. In this paper, we focused our attention on the orthologous protein-coding genes and corresponding coding exons. This approach has obvious drawbacks, as most genomic alignment programs, including Cgaln, are designed to find not only orthologs but also other homologs. Moreover, alignment of non-coding genes and intergenic regions can be misinterpreted in our procedure. However, we expect that this kind of imprecision would not much affect the evaluation of the relative performance of various methods.

**Figure 3:**
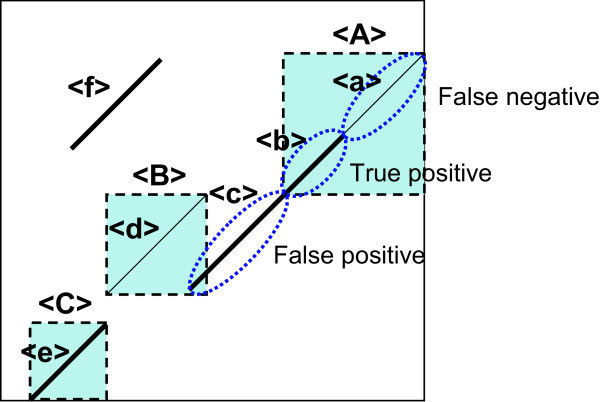
**Calculation of sensitivity and specificity of alignments**. Dotted squares (A), (B), and (C) indicate homologous regions corresponding to homologous exons in an orthologous gene pair (bold square). The thin diagonals indicate the true alignments, while the bold diagonals indicate the observed alignments. Examples of true positive, false positive, and false negative are shown. In this case, region (A) is partly aligned, (B) is not aligned, and (C) is entirely aligned. The regions (b) and (e) are true positives, (a) and (d) are false negatives, and (c) and (f) are false positives.

Figure 3 schematically illustrates a case of alignment results. For a bacterial genome pair, we considered how many homologous base pairs in the reference alignment of orthologous gene pairs are correctly aligned by a given method for calculating sensitivity and specificity. A true positive (*TP*) indicates the number of genomically aligned bases that coincide with those in the reference alignment, a false negative (*FN*) indicates the number of bases in the reference alignment not observed in the genomic alignment, and a false positive (*FP*) indicates the number of genomically aligned bases outside the reference alignment. Thus, the sensitivity (*Sn*) and specificity (*Sp*) are defined as:(7)

For the example shown in Figure 3,(8)

and(9)

For a mammalian chromosomal pair, sensitivity is evaluated as before, in which homologous exons are considered homologous regions. However, it is dificult to evaluate specificity because the biologically significant regions that hold large amounts of the entire genome are not clear. In this examination, we counted the total length of generated HSPs ((b) + (c) + (e) + (f) in Figure 3) as an indicator of specificity, for if the total HSP length from one alignment program is considerably greater than that from other programs with the same sensitivity, the program may be judged to have low specificity. We also evaluated specificity with a human chromosome 20 - mouse chromosome 2 pair. Human chromosome 20 is considered completely orthologous to mouse chromosome 2 [35], and the alignment result of human chromosome 20 and mouse chromosome 2 should coincide with that of human chromosome 20 and the whole genomic sequence of mouse. Therefore, specificity can be evaluated as(10)

where *len*_20-2 _is the total length of HSPs for human chromosome 20 vs. mouse chromosome 2, while *len*_20-*all *_is that for human chromosome 20 vs. whole mouse genome.

### Preparation of data

We obtained the whole-genome sequences of *Escherichia coli *CFT073 (Accession No. NC_004431.1, 5,231,428 bp), *E. coli *K12 (NC_000913.2, 4,639,675 bp), and *Salmonella typhimurium *(NC_003197.1, 4,857,432 bp) from NCBI http://www.ncbi.nlm.nih.gov/. Before applying these genomic sequences to alignment programs, we masked repetitive sequences by MaskerAid [36] with default parameters. We also prepared the whole-genome sequences of human (build hg19) and mouse (build mm9) from the UCSC Genome Browser http://genome.ucsc.edu/. These human and mouse genome sequences were already soft-masked, and we did not use any masking tool. We also downloaded the latest version (KOREF_20090224) of Korean individual (SJK) genomic sequence [37] from the FTP site ftp://ftp.kobic.kr/pub/KOBIC-KoreanGenome/.

Our examination requires reference data on the locations of homologous regions on the input sequences. To obtain such a reference dataset, we first collected sets of orthologous gene pairs. For bacterial genomes, we used MBGD (Microbial Genome Database for Comparative Analysis) [38] for this purpose. MBGD is a database for comparative analysis of microbial genomes, and possesses data on orthologous gene clusters of bacteria. While COG [39] is well known as an orthologous gene database, we preferred MBGD because MBGD is better than COG at clustering orthologous genes in more detail. Data on orthologous gene pairs between human and mouse were obtained from RefSeq http://www.ncbi.nlm.nih.gov/RefSeq/ and Ensembl http://www.ensembl.org/. Next, we aligned corresponding cDNA sequences of all gene pairs by the standard local DP algorithm [24]. Finally, we located each gene on the reference genome. For bacterial pairs, the information on location was obtained from GenBank. For mammalian pairs, we mapped the cDNA sequences on the respective genomic regions by Spaln [40,41]. From 10042 original gene pairs, 9373 pairs (= 18746 genes) could be mapped on the reference genomes for the homologous genomic regions. As 97% (18252) cDNAs were mapped on the reference genomes with 100% identity and the other genes were mapped with more than 95% identity, we regarded these data as valid. By combining the cDNA alignment and the mapping coordinates, we obtained the homologous exonic regions on the genomes.

### Programs used for tests

To compare the performance of our algorithm with other leading programs presently available, we developed the Cgaln program that implements the above-mentioned algorithm in C on a Linux platform. We report on comparisons of the accuracy and computational speed of Cgaln with those of Blastz, AVID, and NUCmer. Blastz is one of the principal pairwise alignment programs for long sequences, and is used as an internal engine of several multiple genomic sequence alignment programs, such as MultiPipMaker [42], TBA, and MultiZ [43]. We examined Blastz with two sets of parameter values; with the default parameter set and with the tuned parameter set (*T *= 2, *C *= 2). The option "*T *= 2" disregards transitions as matches; this speeds up computation but slightly reduces sensitivity. The option "*C *= 2" directs "chain and extend", which helps to reduce false positives. AVID is also a fast and accurate genomic alignment program, but it is a global aligner and not suitable for the alignment of genomes with large rearrangements. We applied AVID only to bacterial genome pairs. NUCmer is a variant of MUMmer 3.0. It clusters the matches of MUMmer 3.0 and tries to align the non-exact regions between the matches by DP.

Although there are other genomic alignment tools, such as CHAOS, LAGAN, SLAGAN, DIALIGN, GLASS, and WABA, they are either too slow to execute or their source codes are not available. All experiments were performed on a 2.0 GHz Core2Quad (64-bit CPU) with 8 GB memory.

## Results

### Parameter tuning

Preliminary examinations indicated that the performance of Cgaln depends strongly on the outcome of BA, and hence a proper choice of parameter values at this level is essential, especially for distantly related genome pairs. We tested Cgaln with various block sizes and *k*-mer sizes, and found that, if appropriate threshold values for "significant" colonies *T*_*col *_are used, there is no remarkable difference in accuracy or computational time in a wide range of block sizes. However, if a block is too large, computation will be slow because a lot of spurious HSPs tend to be generated within block cells. Thus, block sizes between 5,000 bp and 30,000 bp are proven to be almost equally appropriate for both bacterial and mammalian genomes. It is desirable to automatically estimate the optimal *T*_*col *_value for a given set of block sizes, *k*-mer size, and overall divergence between the sequences under comparison. In this study, we first tuned *T*_*col *_for default block size and *k*-mer size (expressed by *T*_*col*-*default*_), and then derived an empirical rule applicable to other block sizes:(11)

where *r*_*b *_is the ratio of the given block size to the default block size.

For a given *k*-mer size, we use the relation:(12)

where *k*_*dif f *_is the difference between the given *k*-mer size and the default *k*-mer size. Although these empirical rules work satisfactorily, a theoretically supported tuning algorithm remains to be established. We set the default value as follows: block size = 10000, *T*_*col*-*default *_= 3000, gap penalty at BA *d *= *T*_*col*-*default*_/15. These parameters can be changed by selecting various options.

### Alignment of bacterial genomes

We first examined the performance of Cgaln in comparison with Blastz, AVID, and NUCmer for two sets of pairwise alignments of bacterial genomes: (E-E) *E. coli *CFT073 vs. *E. coli *K12 (2531 gene pairs), and (E-S) *E. coli *CFT073 vs. *S. typhimurium *(3385 gene pairs).

Table 1 and Table 2 summarize the results of the comparison (E-E) and the comparison (E-S), respectively. For Cgaln, the time spent to make the four kinds of tables is not included. It requires several seconds for a bacterial genome and about 20 minutes for a mammalian whole genome.

**Table 1:** Comparison of performance with *E. coli *CFT073 -*E. coli *K12 pair.

	length (bp)	Sn (%)	Sp (%)	time (s)	memory (MB)
Blastz (default)	5,643,656	97.4	44.4	72	348
Blastz (T = 2 C = 2)	4,035,271	95.3	60.0	22	346
AVID	4,044,527	94.7	59.3	147	2297
NUCmer	1,121,714	26.1	59.2	12	90
Cgaln (-X4000)	4,313,795	96.3	57.2	9	167

Comparison of the performance of Cgaln with other programs for *E. coli *CFT073 vs. *E. coli *K12. The length indicates the total length of generated HSPs. Sn and Sp indicate sensitivity and specificity, respectively. "-X4000" of Cgaln indicates that the threshold *T*_*col *_was set at 4000.

**Table 2:** Comparison of performance with *E. coli *CFT073 - *S. typhimurium *pair.

	length (bp)	Sn (%)	Sp (%)	time (s)	memory (MB)
Blastz (default)	4,952,433	83.0	61.2	73	348
Blastz (T = 2 C = 2)	3,330,116	79.5	84.2	22	347
AVID	3,263,793	78.1	73.8	178	2365
NUCmer	219,651	4.9	69.7	27	90
Cgaln (-X4000)	3,456,219	78.7	81.0	11	167

Comparison of the performance of Cgaln with other programs for *E. coli *CFT073 vs. *S. typhimurium*. "-X4000" of Cgaln indicates the threshold *T*_*col *_was set at 4000.

Blastz with the default parameters produced a lot of spurious alignments, which resulted in high sensitivity but low specificity. With tuned parameters, Blastz improved the specificity and computational time but decreased sensitivity. AVID, a global alignment tool, shows high sensitivity and specificity in the intra-species comparison (E-E). However, it cannot consider inversion and is proven to be insensitive and not specific in interspecies comparisons (E-S). Moreover, AVID consumes a lot of memory, about 2.2 GB. NUCmer is fast and more memory-efficient than Cgaln but is much less sensitive. In (E-E), Nucmer could identify about 82% of all genes, but the coverage of almost all genes was low (~50%), possibly due to the use of long seeds (20-mer), and so it is applicable only to closely related sequence comparisons. Table 1 and Table 2 show that Cgaln is the fastest and second most memory-efficient among these programs, with high accuracy. Compared with Blastz with tuned parameters, Cgaln is twice as fast and more space-efficient, comparably sensitive, but less specific. This inferiority in specificity is due to overalignment at BA. Cgaln with the "-fc" option (see Methods) could improve specificity to a level comparable to that of tuned Blastz. However, it is not always better to use this option, as there is a trade-off between sensitivity and specificity.

### Alignment of mammalian chromosomes

We also compared the performance of Cgaln with that of Blastz and that of NUCmer on two kinds of mammalian homologous chromosome pairs: (H20-M2) human chromosome 20 (63,025,520 bp) vs. mouse chromosome 2 (181,748,087 bp), and (X-X) human chromosome X (155,270,560 bp) vs. mouse chromosome X (166,650,296 bp). The numbers of orthologous gene pairs on (H20-M2) and (X-X) are 278 and 341, respectively.

**Figure 4:**
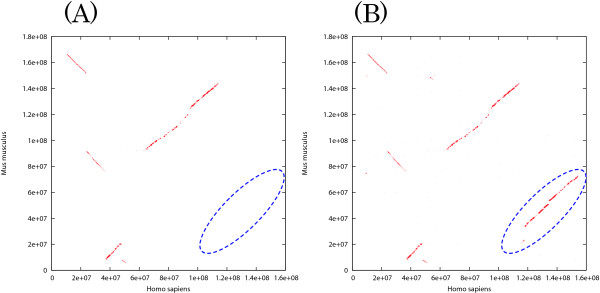
**The alignment results of Blastz between human chromosome X and mouse chromosome X**. The alignment results of Blastz with tuned parameters (C = 2 T = 2) between human chromosome X and mouse chromosome X are represented as dot plots. (A) Non-split sequences. (B) Split sequences. In (A), a homologous segment is lost upon chaining (dotted ellipse).

The results are summarized in Table 3 and Table 4. We applied Blastz to two types of input sequences: (i) intact chromosomal sequences, and (ii) chromosomal sequences split into chunks of 10 Mb with 10 Kb overlaps. The third rows of Table 3 and Table 4 labeled with "*" show the results obtained with the split sequences. The time for splitting and uniting is not included. Blastz with the default parameters resulted in nearly the same accuracy in the cases of (i) and (ii) above. This is not surprising, because Blastz with the default parameters does not chain HSPs. As in the case of bacterial genomes, the results of Blastz with the default parameters were sensitive but not specific, and the computation was slowest among all of the programs and settings examined. The low specificity (56.2%) and the large HSP length (especially in (X-X)) indicate that Blastz generates a lot of repetitious HSPs, although we used masked sequences. The "C = 2" option improved the specificity of Blastz drastically, and the "T = 2" option reduced the computational time with a slight decrease in sensitivity. However, when applied to the intact sequence pair of chromosome X, the tuned Blastz missed some long homologous regions (shown in Figure 4A), which resulted in poor exon coverage (36.5%). With the split sequences, this deficit was not observed (Figure 4B), while specificity declined because filtering by the chaining process was not sufficient. In the case of the chromosomal pair (H20-M2), such a big deficit was not observed, possibly because of the high similarity and the small number of rearrangements between the two chromosomes. The consumed memory of Blastz was large, especially for (X-X) (2.8 GB) when intact chromosomes were examined, regardless of the choice of options. NUCmer was as fast and memory-efficient as Cgaln in examination (H20-M2), but consumed twice as much memory as Cgaln in examination (X-X). Moreover, NUCmer was shown not to be sensitive in the pairwise alignment of mammalian chromosomes.

**Table 3:** Comparison of performance with human chromosome 20 - mouse chromosome 2 pair.

	length (bp)	Sn (%)	Sp_*mam *_(%)	time	memory (GB)
Blastz (default)	18,598,895	85.9	57.5	66 m 40 s	1.6
Blastz (T = 2 C = 2)	16,353,601	85.1	95.5	9 m 45 s	1.6
Blastz* (T = 2 C = 2)	16,665,937	85.3	80.8	12 m 20 s	0.4
NUCmer	1,118,494	5.8	75.2	3 m 55 s	1.0
Cgaln (-X2500 *k *= 11)	13,964,626	79.2	92.3	2 m 14 s	0.8
Cgaln (-X2500 *k *= 12)	15,154,530	81.4	90.6	2 m 16 s	1.1

Performance of alignment programs examined on human chromosome 20 (chr20) vs. mouse chromosome 2 (chr2). The row of Blastz with the symbol "*" refers to the examination on split chromosomal sequences. The threshold *T*_*col *_was set to "-X2500". *Sp*_*mam *_is evaluated as the ratio of the coverage with human chr20 - mouse chr2 to that with the human chr20 - whole-genome sequence of mouse.

**Table 4:** Comparison of performance with human chromosome X - mouse chromosome X pair.

	length (bp)	Sn (%)	time	memory (GB)
Blastz (default)	41,649,203	58.9	117 m 03 s	2.8
Blastz (T = 2 C = 2)	21,094,947	36.5	15 m 21 s	2.8
Blastz* (T = 2 C = 2)	32,684,714	58.0	23 m 22 s	0.4
NUCmer	1,299,148	5.4	4 m 01 s	2.3
Cgaln (-X2500 *k *= 11)	25,675,191	54.4	5 m 56 s	0.9
Cgaln (-X2500 *k *= 12)	27,776,861	56.0	4 m 21 s	1.2

Performance of alignment programs examined on human chromosome X (chrX) vs. mouse chromosome X. The threshold *T*_*col *_was set to "-X2500".

We examined Cgaln with two kinds of *k*-mer spaced seed, 11-mer and 12-mer. Cgaln with 12-mer required more memory than that with 11-mer, but much less than Blastz. Cgaln performed better with the 12-mer spaced seed than with the 11-mer. In fact, several genes were missed with the 11-mer but identified with the 12-mer. With the 11-mer, the number of occurrences of each seed in a block often exceeds the proper range suitable for scoring by Poisson distribution, which we consider the main reason for the lower performance with the 11-mer. It should be noted that with either *k*-mer, Cgaln does not generate much noise like Blastz with default parameters, nor does it cause a big deficit like Blastz with tuned parameters for the intact chromosomal pair (X-X).

However, Cgaln was slightly less sensitive than Blastz because the nucleotide-level coverage of gene pairs in the former was slightly worse in the latter. Scrutinizing the computational processes, we found that the differences in gene coverage between Cgaln and Blastz originate mainly when HSPs are extended with gaps. In fact, we confirmed that the sensitivity of Cgaln was slightly improved when the X-drop-off threshold for gapped extension of HSP or the length-threshold for DP (*T*_*dp*_) was augmented. The slightly smaller total length of HSPs compared with those of Blastz also indicated that Cgaln "underaligns" the sequences. However, we question the significance of the difference in coverage, because Blastz might "overalign" sequences, as the X-drop-off parameter of Blastz is so large that it may improve nominal sensitivity but may align some non-homologous regions [44].

### Application of Cgaln to human and mouse whole genomes

**Figure 5:**
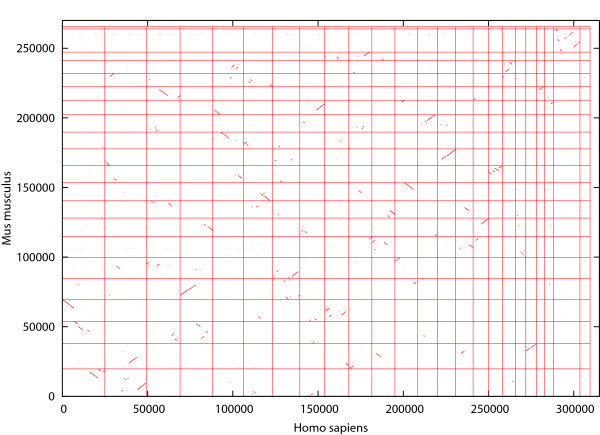
**The result of whole-genome alignment between a human-mouse genome pair**. The result of whole-genome alignment between human and mouse at the block level. The horizontal and vertical lines indicate the delimiters between chromosomes.

We also applied Cgaln to whole genomic sequences of human and mouse to investigate how many homologous genes Cgaln had caught. In this examination, we set a threshold parameter of *T*_*col *_= 3000 and used the 12-mer seed. As the average nucleotide identity levels vary considerably among chromosomal pairs between human and mouse, it might be preferable to change *T*_*col *_values depending on the chromosome pairs. However, for simplicity we used a fixed value in this examination. The computation took 750 m with 1.8 GB of memory on a desktop computer. Cgaln generated HSPs totaling 693,583,236 bp with 73.6% average nucleotide identities. A total of 8897 gene pairs (95%) were identified, including 7229 pairs (77%) with coverage of more than 80% of their entire exon lengths. The total coverage for all genes at the nucleotide level was 70.2%. Dot plots are shown in Figure 5, and indicate that Cgaln can be conveniently used to draw a general view of a homology map between mammalian whole genomes.

### Identification of human individual differences

Recently, Ahn *et al*. reported individual differences between the human reference genome sequence (UCSC build hg18) and a Korean individual genome (SJK) sequence [37]. We applied Cgaln to these genomic sequences to test its ability to identify individual differences. We used *T*_*col *_= 10000 and the 12-mer spaced seed. We also used options "-fc" and "-cs" to filter out repetitive outputs at both BA and NA. The results are summarized in Table 5. As the table shows, the generated HSPs totaled 2,849,038,447 bp including a soft-masked region of 1,421,050,705 bp. The average nucleotide identity was 99.5%. Cgaln identified 3,385,085 SNPs, 98% of those reported in [37] in which 1,702,430 (50%) SNPs were found in masked regions. The SNPs not identified were embedded in entirely masked regions that were omitted in BA. Cgaln also identified 5,932,816 mismatches that were not reported in [37]. Most of them are derived from Ns in the SJK sequence. The others might be derived from the difference between our alignment and that of Ahn *et al*.

**Table 5:** Results of human-human comparison.

strand	length (bp)	length masked (bp)	pi (%)	SNP	SNP masked	mismatch
Forward	2,799,061,317	1,390,502,477	99.6	3,383,112 (98%)	1,704,098 (50%)	3,295,174
Reverse	49,977,130	30,548,228	94.3	35,368 (1.0%)	14,900 (0.4%)	2,691,595
Both	2,849,038,447	1,421,050,705	99.5	3,385,085 (98%)	1,702,573 (50%)	5,932,816

Result of Cgaln with -fc -cs options. "pi" indicates the percent identity of HSPs in total. "SNP" indicates the number of identified SNPs reported in [37], while "mismatch" indicates the number of nucleotide mismatches not reported.

## Discussion

With the dramatic increase in available genomic sequence data, comparative studies using these data are getting wider application, from identification of individual differences in a population to reconstruction of the long-range evolutionary history of genome organizations. Such applications are becoming more and more demanding, in both speed and accuracy, for computational tools that compare whole genomic sequences. Our primary aim in designing Cgaln was to develop an alignment program that can handle large genomic sequences directly on a standalone computer so that it is handy and useful for a wide range of researchers. Our results show that Cgaln is very effective for comparing large genomes, especially of intact chromosomal sequences. Cgaln is several times faster and more memory-efficient than any presently available programs for whole genome alignment; it requires less than 13 hours and 2 GB of memory to align a pair of typical mammalian whole genomes in a single run.

The main feature that distinguishes Cgaln from similar tools is a coarse-grained strategy. This two-step procedure helps to restrict the regions that must be aligned at the nucleotide level; such restriction can drastically reduce the computational time and memory for genomic alignment. While there are several alignment algorithms that adopt preprocessing before detailed alignment to accelerate computation [45,46], the sequences subjected to these algorithms are confined to small genomic regions with high sequence identity. Our algorithm is unique in that it aims at alignment on a much larger scale of more distantly related sequences than other algorithms. Moreover, Cgaln can identify large-scale genomic rearrangements such as inversions, translocations, and duplications at BA. This feature reduces noisy outputs without missing true homologous blocks, while the detected homologous regions can be globally aligned by NA. If one wants simply to take a global view of homology between two genomes, e.g., to infer gross evolutionary events that occurred after their speciation, it is enough to output the BA results obtained at the first stage, which requires much less time than NA.

One issue with the coarse-grained approach is to what extent sequence divergence can be tolerated to achieve a sufficiently sensitive alignment. In this report, we have shown that Cgaln is nearly as sensitive as the best existing programs for the alignment of mammalian genomes. At this moment, we are not confident that the proposed approach is also useful for the alignment of, say, mammalian vs. avian or mammalian vs. fish genomes. However, the problem of insufficient sensitivity could be resolved in several ways, such as by the use of multiple spaced seeds [47], deeper recursive HSP searches, and finding initial seed matches at the translated sequence level rather than at the nucleotide sequence level.

Another issue with the current version of Cgaln is its ability to detect small-scale rearrangements, especially inversions, when it is applied to intra-species genome comparison. Presently, Cgaln can normally detect only inversions larger than the block size (10 Kb by default). To discover smaller rearrangements, we need some modifications. Although the exact solution is a computationally hard problem, we have developed a heuristic method that considers inversions as small as a few hundred bases at the recursive phase applied to inter-HSP regions. Preliminary examination of this modified version of Cgaln on the reference human genome and a Korean individual's genome failed to find additional inversions, including those suggested by [37], some of which were confirmed to be palindromes rather than true inversions. Obviously, it is premature to draw any definitive conclusions from a single example. However, we consider that whole genome alignment may play an essential role in controlling the quality of the outcomes of high-throughput sequencing and analyses.

## Conclusion

Currently, Cgaln is the only program that can align a pair of whole intact genomic sequences of mammals in a single job. Although how to evaluate the accuracy of genomic alignment remains an unsolved problem, our examinations indicate that Cgaln is almost as sensitive and accurate as the best program available today. We believe that Cgaln provides a novel viewpoint for reducing computational complexity, and contributes to other fields of sequence analysis as well as to genomic alignment.

Cgaln needs very little time for BA (about 2 minutes to compare typical mammalian chromosomes), suggesting that Cgaln is capable of extending fast multiple genomic alignment. To this end, we are developing a progressive algorithm that can properly treat rearrangements such as inversions.

## Authors' contributions

RN wrote the program, carried out the experiments, and drafted the manuscript. OG provided the initial conception of Cgaln, suggested ways to improve the code, and helped to improve the manuscript. Both authors read and approved the final manuscript.
